# The contribution of dance to daily physical activity among adolescent girls

**DOI:** 10.1186/1479-5868-8-87

**Published:** 2011-08-04

**Authors:** Jennifer R O'Neill, Russell R Pate, Steven P Hooker

**Affiliations:** 1 Department of Exercise Science, Arnold School of Public Health, University of South Carolina, (921 Assembly Street Suite 212), Columbia, (29208), SC, USA; 2 Prevention Research Center, Arnold School of Public Health, University of South Carolina, (921 Assembly Street), Columbia, (29208), SC, USA

**Keywords:** accelerometer, children, moderate-to-vigorous physical activity, light activity, sedentary behavior

## Abstract

**Background:**

Structured physical activity (PA) programs are well positioned to promote PA among youth, however, little is known about these programs, particularly dance classes. The aims of this study were to: 1) describe PA levels of girls enrolled in dance classes, 2) determine the contribution of dance classes to total moderate-to-vigorous physical activity (MVPA), and 3) compare PA between days with a dance class (program days) and days without a dance class (non-program days).

**Methods:**

Participants were 149 girls (11-18 years) enrolled in dance classes in 11 dance studios. Overall PA was assessed with accelerometry for 8 consecutive days, and girls reported when they attended dance classes during those days. The percent contribution of dance classes to total MVPA was calculated, and data were reduced to compare PA on program days to non-program days. Data were analyzed using mixed models, adjusting for total monitoring time.

**Results:**

Girls engaged in 25.0 ± 0.9 minutes/day of MVPA. Dance classes contributed 28.7% (95% CI: 25.9%-31.6%) to girls' total MVPA. Girls accumulated more MVPA on program (28.7 ± 1.4 minutes/day) than non-program days (16.4 ± 1.5 minutes/day) (p < 0.001). Girls had less sedentary behavior on program (554.0 ± 8.1 minutes/day) than non-program days (600.2 ± 8.7 minutes/day) (p < 0.001).

**Conclusions:**

Dance classes contributed a substantial proportion (29%) to girls' total MVPA, and girls accumulated 70% more MVPA and 8% less sedentary behavior on program days than on non-program days. Dance classes can make an important contribution to girls' total physical activity.

## Background

Helping youth achieve the current physical activity guideline of at least 60 minutes of daily moderate-to-vigorous physical activity (MVPA) is a key public health objective for the 21^st ^century [1]. Structured physical activity programs are major avenues for providing physical activity to youth, and as such, they are a recommended strategy for the promotion of physical activity [1-3]. Structured physical activity programs are organized activities that are typically planned and occur within a specific setting [4]. These programs include physical education classes, organized sports, activity classes or lessons, and after-school programs.

Although structured physical activity programs are well positioned to assist youth in meeting the physical activity guideline, little is known about these programs. Specifically, there is limited knowledge of the overall physical activity levels of program participants and the contribution of these programs to overall physical activity. In addition, very few structured physical activity programs have been studied previously using objective measurement of physical activity by accelerometry. Only one study [5] has used accelerometry to examine the contribution of structured physical activity programs to total daily physical activity. Wickel and Eisenmann [5] found that among 6- to 12-year-old boys, youth sport and physical education classes contributed approximately 23% and 11% to their daily MVPA, respectively.

Dance classes are an important example of structured physical activity programs, because dance is a highly prevalent type of physical activity among adolescent girls [6-8]. Given the steep decline in girls' physical activity levels during adolescence [9-13], there is a need to study dance participation in adolescent girls. Further, the overall physical activity levels of girls who participate in dance classes are unknown, as is the contribution of dance classes to girls' overall physical activity levels. Accordingly, the objectives of this study were 1) to describe the overall physical activity levels of girls who participate in structured dance classes, 2) to determine the contribution of structured dance classes to total light, moderate, vigorous, and MVPA, and 3) to compare physical activity between days with a dance class (program days) and days without a dance class (non-program days).

## Methods

### Study Design

This study employed a cross-sectional design in describing overall physical activity levels in girls who participate in structured dance classes. Each aim was addressed using a distinct methodology and design. Data were collected in a sample of girls registered for lessons in dance studios in Columbia, South Carolina. To be eligible, a dance studio was required to offer at least one ballet, jazz, or tap class per week to students aged 11 years and older. This protocol was designed to measure girls' physical activity via accelerometry over an eight-day period, and to link those data with the structured dance classes each girl attended during that period. In addition, girls' self-report of their structured dance classes allowed for determination of a program day (day with a dance class) and a non-program day (day without a dance class), for the comparison of physical activity levels.

### Participants

Participants were girls (ages 11 to 18 years) enrolled in dance classes at dance studios in Columbia, South Carolina. Forty-three dance studios, defined as commercial facilities whose primary business is to provide dance instruction, were identified using local published and electronic telephone books. The director of each dance studio was contacted by telephone to determine the studio's eligibility status. Twenty-three dance studios were eligible and were invited to participate. Of the 23 eligible dance studios, 11 directors agreed to participate, and those directors provided the schedules of the ballet, jazz, and tap classes that were offered to students aged 11 years and older. In each of the 11 dance studios, one to four classes (based on the studio size and the styles of dance offered) were selected for measurement, from which girls were recruited. Classes were not selected randomly; in small studios where there was only one eligible class, that class was chosen. In larger studios, convenience samples were taken that allowed for a variety of dance styles across age ranges. Girls were eligible to participate if they were enrolled in these dance classes and met the age criteria, which was a total of 212 girls. Of these students, 149 (70.3%) agreed to participate. Written informed consent was provided by each student's parent or guardian and informed assent was given by each student (if < 18 years) prior to collection of data. The study was approved by the University of South Carolina Institutional Review Board.

### Objective Measurement of Physical Activity

ActiGraph accelerometers (Model 7164, Pensacola, FL) were used to measure time spent in sedentary behavior, light, moderate, and vigorous physical activity, and combined MVPA. The ActiGraph is a reliable [14] and valid method for assessing children's and adolescent's physical activity, both in laboratory and field settings [15,16]. The cut-points established by Treuth and colleagues for use in adolescent girls aged 13 to 14 years were used to determine intensity levels [17]. Accelerometers were initialized to save data in 30-second intervals, in accordance with the procedures of Treuth et al. [17]. Accordingly, intensities of physical activity were operationally defined as: sedentary (< 50 counts/30 seconds), light (51-1499 counts/30 seconds), moderate (1500-2600 counts/30 seconds), vigorous physical activity (> 2600 counts/30 seconds), and MVPA (≥ 1500 counts/30 seconds) [17].

Accelerometers provide activity intensity counts which are associated with the corresponding date and time stamp. However, accelerometers do not detect the type of activity performed at any given time. Therefore, to estimate the amount of physical activity in structured dance classes, it was necessary for girls to report the days and times (e.g., Wednesday 5:00 PM - 6:30 PM) they participated in structured dance classes during the week when the accelerometer was worn. Girls reported this information on a written survey.

### Measurement Protocol

Overall physical activity was assessed with accelerometry for eight days. A research assistant placed accelerometers on participants approximately 10 minutes before the beginning of the dance class. Accelerometers were attached to an elastic belt and worn over the right hip. Participants wore the accelerometers during the entire class and continued to wear them for the following seven days. They were given instructions to wear the accelerometer during all waking hours, except when swimming or bathing. Participants wore the accelerometers to the same dance class the following week, and the accelerometers were removed by the research assistant at the end of the class. Upon collection of the accelerometers, activity counts were downloaded and saved on a computer for data reduction and analysis. In addition to the accelerometer collection, girls completed a written survey in which they reported the days and times they attended structured dance classes during the past week.

### Data Reduction

#### Overall physical activity

An established method of accelerometer data reduction is to concatenate data from the first day and last day of data collection when those days are the same day of the week [18]. In this study, the accelerometers were put on (day 1) and taken off (day 8) on the same day of the week, and for data reduction, a single day of data was created by combining the last part of day one and the first part of day eight. Periods of 60 minutes or more of zeros were considered to be non-wear time and were excluded from the analysis. Adherent days were those with a minimum of eight hours per weekday and six hours per weekend day of monitoring time. Missing accelerometer data on non-adherent days were replaced via imputation using the expectation maximization algorithm, based on the methods of Catellier et al. [18]. Data were also reduced without imputation; however, there were no differences between the mean physical activity levels, therefore, the imputed means are presented.

#### Contribution of dance classes to total light, moderate, vigorous, and MVPA

Several steps were used to determine the contribution of dance classes to total light, moderate, vigorous, and MVPA. This analysis was conducted separately for light, moderate, vigorous, and MVPA, but for simplicity, only MVPA will be included in this section. First, total MVPA was calculated. As in the previous analysis, data from day one and day eight were combined, and periods of 60 minutes or more of zeros were considered to be non-wear time and were excluded from the analysis. Adherent days were those with a minimum of eight hours per weekday and six hours per weekend day of monitoring time. To be included in this analysis, girls were required to have a minimum of three weekdays and one weekend day. For each of these qualifying days, the amount of time (minutes) spent in MVPA was calculated. For each girl, MVPA was divided by the corresponding number of qualifying days to obtain the average minutes per day of total MVPA. Second, MVPA in dance classes was determined. For each of the qualifying days, accelerometer counts collected during the reported duration of each dance class were segmented from the raw accelerometer data file. The amount of time (minutes) spent in MVPA during dance classes was calculated. If a girl did not report participation in a dance class on a qualifying day, MVPA during dance class was zero. For each girl, MVPA in dance classes was divided by the corresponding number of qualifying days to obtain the average minutes per day of MVPA in dance classes. Third, the percent contribution of dance classes to total MVPA was calculated from minutes per day of MVPA in dance classes and minutes per day of total MVPA.

#### Program days vs. non-program days

Data were also reduced to compare physical activity on program days to physical activity on non-program days. For this analysis, only weekdays with eight or more hours of monitoring time were included. Because the first day and last day of accelerometer wear (day one and day eight) were not full days of wear, these days were excluded. All eligible days were used in the analysis, with the minimum of one program day and one non-program day. The amount of time (minutes) spent in sedentary, light, moderate, vigorous, and MVPA were calculated separately for the program and the non-program days.

### Anthropometric Measures

Height and weight measurements were conducted in a private setting. Height and weight were assessed objectively using a portable stadiometer measured to the nearest 0.1 cm (Shorr Productions; Olney, MD) and an electronic scale measured to the nearest 0.1 kg (model 770; Seca, Hamburg, Germany). The average of two measurements was used. Body mass index (BMI) was calculated and expressed as body mass (kg) divided by height (m^2^), and BMI was converted to BMI percentiles using the CDC Growth Charts [19].

### Additional Variables

Participants' date of birth and race/ethnicity were reported by parents on the consent form. To describe the sample, participants self-reported information about their dance participation. This included the starting age of dance instruction, dance styles ever studied, number of dance classes taken per week, hours of rehearsal per week, and participation in competitive or company dance. The number of classes per week, rehearsal hours per week, and competitive or company dance were in reference to current participation (i.e., present time).

### Statistical Analyses

All data were analyzed using generalized linear mixed models (PROC MIXED). For the purpose of comparing program days and non-program days, least squares means were used, and models were adjusted for total monitoring time. Means and standard errors were calculated. SAS software (version 9.2; SAS Institute, Cary, NC) was used for all statistical analyses. Statistical significance was set at *P *< 0.05.

## Results

A total of 149 girls participated in the study. Eleven girls were excluded due to accelerometer malfunctions, and four were excluded for incomplete data, leaving 134 included in the data analyses. Due to the three analytical methods, there were three analytic samples; the descriptive characteristics of the three samples are presented in Table 1. The mean age was 14.6 ± 1.9 years, and over 80% of the participants were Caucasian. There are many styles of dance, and girls reported participating in the following styles during the previous week: ballet, jazz, tap, contemporary, hip hop, theatrical jazz/musical theatre, lyrical, baton, character, belly dance and ballroom.

**Table 1:** Descriptive characteristics of girls enrolled in structured dance classes.

		Sample 1 n = 134		Sample 2 ‡ n = 101		Sample 3 ¶ n = 76
	**n**	**Mean** **(SD) or %**	**n**	**Mean** **(SD) or %**	**n**	**Mean** **(SD) or %**

Age (yr)	134	14.6 ± 1.9	101	14.5 ± 1.9	76	14.7 ± 2.0
Race/ethnicity, %						
Caucasian	108	80.6%	82	81.2%	62	81.6%
African American	11	8.2%	6	5.9%	7	9.2%
Other **	15	11.2%	13	12.9%	7	9.2%

Height (cm)	134	159.8 ± 7.2	101	159.1 ± 7.4	76	159.8 ± 8.0
Weight (kg)	134	51.8 ± 10.3	101	50.8 ± 10.8	76	52.1 ± 9.4
BMI (kg/m^2^)	134	20.2 ± 3.5	101	20.0 ± 3.7	76	20.3 ± 2.7
BMI Percentile	134	51.0 ± 27.0	101	49.0 ± 27.6	76	53.7 ± 25.3
Age began dance training (yr)	134	4.4 ± 2.7	101	4.5 ± 2.8	76	4.3 ± 2.4

Dance training (yr)	134	10.2 ± 3.2	101	10.0 ± 3.2	76	10.4 ± 3.0
Dance styles studied †	134	5.9 ± 1.9	101	6.0 ± 1.9	76	5.8 ± 1.7
Dance classes/wk * §	134	6.2 ± 2.6	101	6.6 ± 2.7	76	5.9 ± 1.8
Rehearsal/wk (h) *	134	6.1 ± 5.4	101	6.5 ± 5.9	76	5.7 ± 4.1
Company or competitive dance (%) *	105	78.4%	77	76.2%	61	80.3%

SD, standard deviation; BMI, body mass index.

‡ Sample 2: Girls with ≥ 3 weekdays and ≥ 1 weekend day.

¶ Sample 3: Girls with ≥ 1 program and 1 non-program day.

** Sample 1: Asian (n = 5), Hispanic (n = 1), Multi-racial/Other (n = 5), Not Reported (n = 4).

† Dance styles ever studied: ballet, jazz, tap, contemporary, hip hop, theatrical jazz/musical theatre, lyrical, baton, character/folk, ballroom, African, Indian, Irish, social dance, clogging, and belly dance.

* Referred to current participation (i.e., present time).

§ The average dance class length was 71.1 ± 18.1 minutes.

### Overall physical activity

The analysis sample for overall physical activity included 134 girls. The means for the overall physical activity intensities are presented in Table 2. Girls obtained an average of 25.0 minutes per day of MVPA, 271.1 minutes per day of light physical activity, and 535.0 minutes per day of sedentary behavior.

**Table 2:** Overall physical activity, minutes per day, among girls enrolled in dance classes, (n = 134).

	**Mean **± **SE**	95% CI
Sedentary	535.0 ± 8.7	517.8-552.1
Light	271.1 ± 5.0	261.1-281.0
Moderate	18.0 ± 0.6	16.8-19.2
Vigorous	7.0 ± 0.3	6.4-7.7
MVPA	25.0 ± 0.9	23.3-26.7

MVPA, moderate-to-vigorous physical activity; SE, standard error;

CI, confidence interval.

### Contribution of dance classes to total light, moderate, vigorous, and MVPA

A total of 33 girls were excluded from this analysis for not having the required amount of qualifying days, resulting in an analysis sample of 101 girls. Excluded girls did not differ from other girls for any of the demographic variables or overall physical activity; however, excluded girls had fewer dance classes per week compared to others (4.9 ± 1.8 vs. 6.6 ± 2.7, *P *< .001). The average reported dance class length was 71.1 ± 18.1 minutes. During structured dance classes, girls accumulated an average of 39.4 minutes per day of light physical activity and 7.1 minutes per day of MVPA (Table 3). Dance classes contributed 15.4%, 26.7%, 39.6%, and 28.7% of the girls' total light, moderate, vigorous, and MVPA, respectively.

**Table 3:** Contribution of dance classes to total light, moderate, and vigorous physical activity and MVPA (n = 101).

	Dance (min/d)		Total (min/d)		Percent Contribution	
	**Mean **± **SE**	**95% CI**	**Mean **± **SE**	**95% CI**	**Mean **± **SE**	**95% CI**

LPA	39.4 ± 1.9	35.7 - 43.1	266.7 ± 6.4	254.0 - 279.4	15.4% ± 0.8%	13.8% - 17.0%
MPA	4.4 ± 0.3	3.8 - 4.9	17.3 ± 0.8	15.8 - 18.8	26.7% ± 1.4%	23.8% - 29.5%
VPA	2.7 ± 0.2	2.3 - 3.0	7.0 ± 0.4	6.2 - 7.8	39.6% ± 2.2%	35.4% - 43.9%
MVPA	7.1 ± 0.4	6.2 - 7.9	25.2 ± 1.1	23.0 - 27.5	28.7% ± 1.4%	25.9% - 31.6%

LPA, light physical activity; MPA, moderate physical activity; VPA, vigorous physical activity; MVPA, moderate-to-vigorous physical activity; SE, standard error; CI, confidence interval.

### Program day vs. non-program day

To be included in this analysis, girls needed to have at least one program day and one non-program day. From the first sample of 134 girls, 13 girls were excluded because they had less than two eligible days, 10 girls were excluded because they did not have any program days (on the eligible days), and 35 girls were excluded because they did not have any non-program days (i.e., they attended dance class on every eligible day). Therefore, the sample consisted of 76 girls. Excluded girls did not differ from other girls for any of the demographic variables or overall sedentary, light, or moderate physical activity; however, excluded girls had a slightly greater percentage of vigorous (1.0% ± 0.4% vs. 0.8% ± 0.5%, p = 0.01) and MVPA (3.3% ± 1.0% vs. 2.8% ± 1.3%, p = 0.03) compared to the other girls. The comparison of physical activity intensities between program days and non-program days are presented in Table 4. There were no significant differences in the total monitoring time between the two days (program day: 854.5 ± 12.0 minutes; non-program day: 880.0 ± 15.0 minutes; p = 0.13). After controlling for total monitoring time, girls accumulated significantly more minutes of MVPA on program days (28.7 ± 1.4 minutes per day) than non-program days (16.4 ± 1.5 minutes per day) (p < 0.001). Girls had significantly fewer minutes of sedentary behavior on program days (554.0 ± 8.1 minutes per day) than non-program days (600.2 ± 8.7 minutes per day) (p < 0.001). Girls engaged in significantly more light, moderate, and vigorous physical activity on program days than non-program days (p < 0.001).

**Table 4:** Program days vs. non-program days, minutes/day (mean ± SE), (n = 76).†

	Program Days	Non-Program Days	p-value
Sedentary	554.0 ± 8.1	600.2 ± 8.7	< 0.001
Light	282.0 ± 7.3	248.0 ± 7.9	< 0.001
Moderate	20.8 ± 1.0	13.0 ± 1.1	< 0.001
Vigorous	7.9 ± 0.5	3.4 ± 0.6	< 0.001
MVPA	28.7 ± 1.4	16.4 ± 1.5	< 0.001
Monitoring Time	854.5 ± 12.0	880.0 ± 15.0	0.13

MVPA, moderate-to-vigorous physical activity; SE, standard error.

† Adjusted for total monitoring time.

In addition, we analyzed the physical activity data from the girls with zero non-program days (i.e., they attended dance class on every eligible day). Overall, these girls obtained an average of 28.9 minutes per day of MVPA, 9.0 minutes per day of vigorous physical activity, and 292.5 minutes per day of light physical activity (Table 5). They engaged in more vigorous physical activity and MVPA per day than girls in Sample 1. During structured dance classes, these girls accumulated an average of 54.5 minutes per day of light physical activity and 9.4 minutes per day of MVPA (Table 6), which was significantly more than girls in Sample 1. Dance classes contributed 20.5%, 31.2%, 42.6%, and 33.1% of the girls' total light, moderate, vigorous, and MVPA, respectively. This sub-sample of girls had a higher percent contribution of dance classes to total light, moderate, vigorous, and MVPA than girls in Sample 1.

**Table 5:** Overall physical activity for girls with zero non-program days, minutes/day (mean ± SE), (n = 35).

	**Mean **± **SE**	95% CI
Sedentary	540.4 ± 14.2	511.3-568.8
Light	292.5 ± 8.3	275.6-309.3
Moderate	19.8 ± 1.1	17.7-22.0
Vigorous	9.0 ± 0.7	7.7-10.4
MVPA	28.9 ± 1.5	25.8-32.0

MVPA, moderate-to-vigorous physical activity; SE, standard error;

CI, confidence interval.

**Table 6:** Contribution of dance classes to total light, moderate, and vigorous physical activity and MVPA for girls with zero non-program days (n = 31).

	Dance (min/d)		Total (min/d)		Percent Contribution	
	**Mean **± **SE**	**95% CI**	**Mean **± **SE**	**95% CI**	**Mean **± **SE**	**95% CI**

LPA	54.5 ± 2.5	49.4 - 59.6	278.3 ± 11.1	255.5 - 301.0	20.5% ± 1.3%	18.0% - 23.1%
MPA	5.6 ± 0.5	4.5 - 6.6	18.6 ± 1.2	16.2 - 21.0	31.2% ± 2.4%	26.3% - 36.0%
VPA	3.8 ± 0.3	3.2 - 4.4	9.4 ± 0.7	8.0 - 10.7	42.6% ± 3.5%	35.6% - 49.7%
MVPA	9.4 ± 0.8	7.8 - 10.9	29.2 ± 1.8	25.6 - 32.8	33.1% ± 2.3%	28.3% - 37.9%

LPA, light physical activity; MPA, moderate physical activity; VPA, vigorous physical activity; MVPA, moderate-to-vigorous physical activity; SE, standard error; CI, confidence interval.

## Discussion

This was the first study to describe the physical activity levels of girls who were enrolled in structured dance classes using an objective measure of physical activity. Activity performed in structured dance classes accounted for a substantial proportion (29%) of their total weekly MVPA. Most notably, girls obtained 70% more total MVPA and 8% less sedentary behavior on program days (i.e., participation in dance class), than non-program days. Therefore, these novel findings provide strong evidence of the important contribution that dance classes can make to girls' total physical activity.

The present study used accelerometry to measure physical activity, which has been done very rarely to examine the contribution of structured physical activity programs to total physical activity. Although several previous studies [5,20-22] have used accelerometry to examine physical activity *during *structured physical activity programs, to the best of our knowledge, only one study [5] has used accelerometry to examine the contribution of structured physical activity programs to total physical activity. Organized sports and physical education contributed 23% and 11% to boys' total daily MVPA, respectively [5]. These are lower than the contribution of dance classes in the present study (29%). Other studies have examined the contribution of structured physical activity programs to total physical activity, but they utilized different methodologies to assess physical activity [23,24]. For example, Katzmarzyk and Malina administered a 3-day activity diary to 12- to 14-year-old sport participants and found that youth sport contributed to 60% of their daily moderate to vigorous energy expenditure [24]. Morgan et al. [23] used pedometers in 1^st ^through 6^th ^graders and reported that the least, moderately active, and most active children obtained approximately 20%, 9%, and 16% of their total steps per day during physical education classes. The differences in these estimates are most likely attributable to the differences in physical activity assessments. However, it is important to recognize the advantages of accelerometry over other physical activity measurements: its ability to measure objectively, assess intensity, and provide activity counts with corresponding date and time stamps. Nonetheless, the present study supports previous findings that structured physical activity programs provide youth with a substantial proportion of MVPA to total MVPA.

A key finding of this study was that girls accumulated 70% more MVPA on program days (i.e., participation in dance class) than on non-program days. Therefore, the additional amount of MVPA obtained on program days was not maintained on non-program days. Further, girls in this study had 46.2 fewer minutes of sedentary behavior on program days compared to non-program days. These findings are consistent with those of Wickel and Eisenmann [5] who found that 6- to 12-year-old boys engaged in more MVPA and less sedentary behavior on a sport day compared to a non-sport day. The present findings are also similar to those of Dale et al. [25], who reported that when school physical activity opportunities were restricted, children did not compensate by increasing their activity during the after-school hours. These findings provide compelling evidence of the potential importance of dance classes for increasing MVPA and reducing sedentary behavior in adolescent girls.

Although a substantial proportion of girls' total weekly MVPA was attributable to dance classes, girls in this study were observed to have physical activity levels approximately the same as girls in other studies that objectively measured physical activity. Girls in this study engaged in an average of 25 minutes of MVPA per day, which is consistent with sixth-grade girls in the Trial of Activity for Adolescent Girls (TAAG) study who accumulated 23.7 minutes of MVPA per day [26]. The physical activity estimates for TAAG were also reported by geographic location, including South Carolina, and that sub-sample of girls obtained 20.8 minutes of MVPA per day [26]. Both the current study and TAAG used the accelerometer cut-points developed by Treuth et al. [17]. The present findings are also similar to those of 12- to 15-year-old girls in the National Health and Nutrition Examination Survey (NHANES) who obtained 24.6 minutes of MVPA per day [27]. In contrast, the amount of daily MVPA of girls in this study was slightly higher than that of 12-year-old girls in the UK [28] and 16- to 19-year-old girls in NHANES [27] who engaged in 18.3 and 19.6 minutes of MVPA per day, respectively. The MVPA cut-point in the UK study [28] was higher than the current study, whereas the MVPA cut-point in NHANES [27] was lower than the current study, which could potentially explain the differences in estimates. It is also important to note that in this study, the standard deviation was 10.1 minutes of MVPA per day, which indicates high inter-individual variability, which is consistent with the findings of Pate et al. [26]. These findings together indicate the need to increase overall physical activity levels of adolescent girls, as they, on average, are not achieving the physical activity guideline of at least 60 minutes of daily MVPA [1].

This study has strengths and limitations that should be noted. An important strength of this study was the objective measurement of physical activity and sedentary behavior via accelerometry. A second strength was the inclusion of a variety of dance styles from 11 dance studios. Another strength was the ability to measure physical activity on both program and non-program days. Limitations were that the accelerometer cut-points were developed for 13- to 14-year-old girls and were applied to girls ages 11 to 18 years, and the cut-points were not specifically developed for dancing. This may have resulted in an underestimation of physical activity from arm movement and its associated energy expenditure. Another limitation was the self-report of dance class schedules, which may be subject to bias and recall limitations. Lastly, because data were collected in one metropolitan area and mostly with Caucasian girls, they may not be generalizable to other populations. However, the sample size allowed us to test multiple hypotheses.

## Conclusion

In summary, our findings demonstrate the importance of dance classes to girls' physical activity levels. We found that dance classes contributed a substantial proportion (29%) of MVPA to girls' total weekly MVPA, and girls accumulated 70% more MVPA and 8% less sedentary behavior on dance class days than on non-dance class days. Thus, the additional minutes of MVPA on dance class days were not maintained on non-dance class days. Therefore, dance classes can play a critical role by providing health-enhancing physical activity to adolescent girls, and can assist them in meeting the current physical activity guideline.

## Competing interests

The authors declare that they have no competing interests.

## Authors' contributions

JRO conceived of and designed the study, acquired the data, analyzed and interpreted the data, and drafted the manuscript. RRP provided critical input during data analysis and manuscript development. The co-authors (RRP and SPH) participated in the interpretation of data and critical revision for important intellectual content. All authors read and approved the final manuscript.
